# Supplementary material for: Risk factors for sacrococcygeal pilonidal sinus: a systematic review and meta-analysis supplemented by genetic causal assessment
Source: Front Surg. 2026 Jan 7;12:1718589. doi: 10.3389/fsurg.2025.1718589 (PMC12819706; doi:10.3389/fsurg.2025.1718589)
Supplement: Supplementary file 2 [file Datasheet2.zip › Supplementary Data 2/MR_pipeline_p5e-8/forest_plots/ukb-d-1747_2.finngen_R12_L12_PILONIDALCYST.pdf]

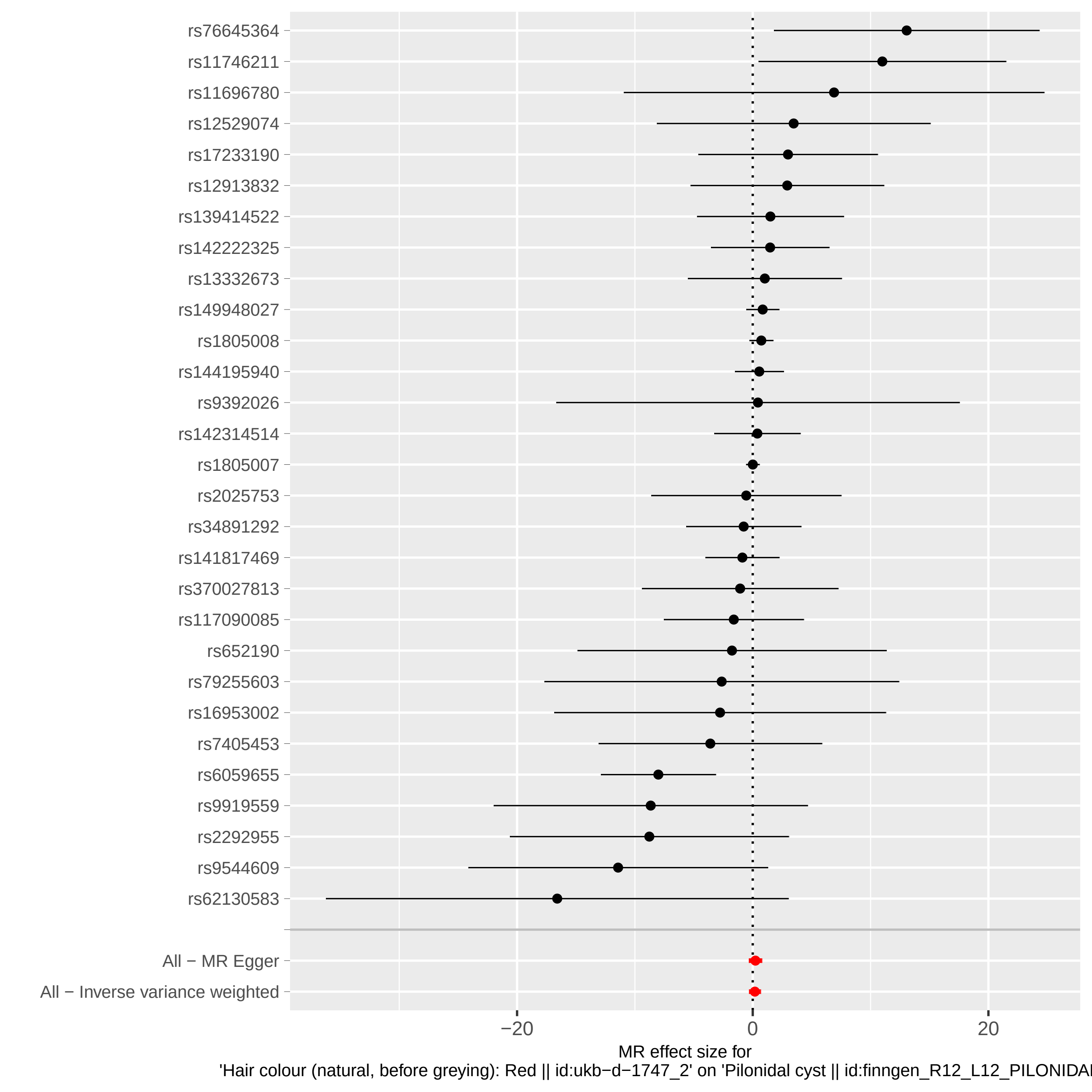

rs76645364
rs11746211
rs11696780
rs12529074
rs17233190
rs12913832
rs139414522
rs142222325
rs13332673
rs149948027
rs1805008
rs144195940
rs9392026
rs142314514
rs1805007
rs2025753
rs34891292
rs141817469
rs370027813
rs117090085
rs652190
rs79255603
rs16953002
rs7405453
rs6059655
rs9919559
rs2292955
rs9544609
rs62130583
All − MR Egger
All − Inverse variance weighted
−20
0
20
MR effect size for
'Hair colour (natural, before greying): Red || id:ukb−d−1747_2' on 'Pilonidal cyst || id:finngen_R12_L12_PILONIDAL
